# Internal tandem duplication mutations in the tyrosine kinase domain of FLT3 display a higher oncogenic potential than the activation loop D835Y mutation

**DOI:** 10.1007/s00277-018-3245-5

**Published:** 2018-01-25

**Authors:** Alissa Marhäll, Florian Heidel, Thomas Fischer, Lars Rönnstrand

**Affiliations:** 1 Division of Translational Cancer Research, Department of Laboratory Medicine, Lund University, Medicon Village, Lund, Sweden; 2 Lund Stem Cell Center, Department of Laboratory Medicine, Lund University, Lund, Sweden; 3 Internal Medicine II, Hematology and Oncology, University Hospital Jena, Jena, Germany; 4 Department of Hematology and Oncology, Otto-von-Guericke University Medical Center, Magdeburg, Germany; 5 Division of Oncology, Skåne University Hospital, Lund, Sweden

**Keywords:** FLT3, Internal tandem duplication, ITD, Receptor tyrosine kinase, Acute myeloid leukemia

## Abstract

Acute myeloid leukemia (AML) remains the most common form of acute leukemia among adults and accounts for a large number of leukemia-related deaths. Mutations in FMS-like tyrosine kinase 3 (FLT3) is one of the most prevalent findings in this heterogeneous disease. The major types of mutations in FLT3 can be categorized as internal tandem duplications (ITD) and point mutations. Recent studies suggest that ITDs not only occur in the juxtamembrane region as originally described, but also in the kinase domain. Although the juxtamembrane ITDs have been well characterized, the tyrosine kinase domain ITDs have not yet been thoroughly studied due to their recent discovery. For this reason, we compared ITD mutations in the juxtamembrane domain with those in the tyrosine kinase domain, as well as with the most common activating point mutation in the tyrosine kinase domain, D835Y. The purpose of this study was to understand whether it is the nature of the mutation or the location of the mutation that plays the main role in leukemogenesis. The various FLT3 mutants were expressed in the murine pro-B cell line Ba/F3 and examined for their capacity to form colonies in semisolid medium. The size and number of colonies formed by Ba/F3 cells expressing either the internal tandem duplication within juxtamembrane domain of the receptor (JMD-ITD) or the tyrosine kinase domain (TKD)-ITD were indistinguishable, while Ba/F3 cells expressing D835Y/FLT3 failed to form colonies. Cell proliferation and cell survival was also significantly higher in TKD-ITD expressing cells, compared to cells expressing D835Y/FLT3. Furthermore, TKD-ITD is capable of inducing phosphorylation of STAT5, while D835Y/FLT3 fails to induce tyrosine phosphorylation of STAT5. Other signal transduction pathways such as the RAS/ERK and the PI3K/AKT pathways were activated to the same level in TKD-ITD cells as compared to D835Y/FLT3 expressing cells. Taken together, our data suggest that TKD-ITD displays similar oncogenic potential to the JMD-ITD but a higher oncogenic potential than the D835Y point mutation.

## Introduction

FMS-like tyrosine kinase 3 (FLT3) is a member of the class III family of receptor tyrosine kinases. FLT3 receptor is composed of an extracellular ligand-binding domain consisting of five immunoglobulin-like domains, a transmembrane domain, a juxtamembrane domain, and an intracellular kinase domain split into two parts by the so-called kinase insert. Upon binding of its ligand, FLT3 ligand (FL), receptors dimerize and become activated. The activated receptor promotes proliferation, survival, and differentiation of early myeloid and lymphoid precursors [1, 2]. Acute myeloid leukemia (AML) is characterized by clonal expansion of myeloid progenitor cells. Up to 30% of patients with AML harbor a mutation in FLT3 thus making it the most frequently mutated gene [3, 4].

The first mutation in FLT3 identified by Nakao and co-workers in 1996 was the so-called internal tandem duplication within juxtamembrane domain of the receptor (JMD-ITD) and for many years, this was thought to be the exclusive location of ITD mutations [4]. Thirteen years later, it was revealed that around 30% of ITDs are located within the tyrosine kinase domain (TKD) region [5]. A few years after the discovery of JMD-ITD, a second type of mutation was discovered in the activation loop of the FLT3 kinase domain, a point mutation at aspartate 835, which is present in around 10% of the AML patients [6]. AML patients with FLT3-ITD are characterized by early relapse and decreased survival in comparison to those expressing wild-type FLT3. Patients harboring ITD mutations within the TKD (TKD-ITD) have a worse survival prognosis in comparison to those with JMD-ITD, the reason for which is still unknown [7]. The length of ITD can be as short as 3 base pairs and up to 400 base pairs and always occurs in frame. Recently, using different JMD-ITD and TKD-ITD mutants, it has been shown that location of the ITD in FLT3 influences its sensitivity to tyrosine kinase inhibitors as well as disease progression in mice [8]. However, the mechanisms by which JMD-ITD and TKD-ITD differ in their oncogenic potentials remain unknown.

Despite the use of new generation kinase inhibitors, certain FLT3 mutations are still associated with high risk of relapse and poor survival. A better understanding of how individual FLT3 mutations contribute to higher risk of relapse and poor survival will help in the design of more effective treatments. Although the JMD-ITD has been well characterized, the TKD-ITDs have due to their recent discovery not yet been thoroughly studied. In this study, we compare ITD mutations in the JMD and TKD, as well as the point mutation located in the TKD, D835Y. The purpose of this study was to investigate whether it is the nature of the mutation or its location that plays a driving role in leukemogenesis. We observed that TKD-ITD mutations have stronger transforming potential that the D835Y mutation.

## Material and methods

### Cell culture and transfection

Ba/F3 cells expressing JMD-ITD and TKD-ITD constructs were provided by courtesy of T. Fischer and F. H. Heidel [8]. Ba/F3 cells expressing wild-type (WT) FLT3, EV, MIG, and D835Y were stably transfected using retroviral transfection system [9]. The cells were cultured in RPMI 1640 medium (Hyclone, Thermo Scientific, Waltham, MA) supplemented with 10% heat-inactivated fetal bovine serum (Life Technologies, Carlsbad, CA), 10 ng/ml recombinant murine interleukin 3 (IL3) and 100 units/ml penicillin, and 100 μg/ml streptomycin. The cells were grown at 37 °C in a humidified atmosphere containing 5% CO_2_.

### Immunoprecipitation and Western blotting

Prior to stimulation with FLT3 ligand (100 ng/ml, 5 min) Ba/F3 cells were starved for 4 h in absence of cytokines and serum. After stimulation, cells were washed with cold PBS before lysis. Cell lysates were prepared by extracting proteins with lysis buffer [40 mM Tris-HCl (pH 8.0), 120 mM NaCl, 0.1% Nonidet-P40] supplemented with protease inhibitors. For immunoprecipitation, 1 μg of antibody per ml of cell lysate was used. Immunoprecipitated proteins were separated by SDS-PAGE and transferred to PVDF membranes. Membranes were blocked with 5% non-fat dry milk in Tris-buffered saline and incubated with primary antibodies for overnight at 4 °C. Blots were developed with a peroxidase-conjugated secondary antibody, and proteins were visualized by enhanced chemiluminescence (ECL) procedures (Amersham, Arlington Heights, IL), using the manufacturer’s protocol. Antibodies used in this study: FLT3 (rabbit polyclonal antibody produced in-house) [10], FLAG (Sigma Aldrich), 4G10 (Millipore), ERK2 (Santa Cruz Biotechnology), pERK1/2 (Santa Cruz Biotechnology), AKT (Santa Cruz Biotechnology), pAKT (Cell Signaling Technology), p38 (BD Transduction Laboratories), pp38 (BD Transduction Laboratories), STAT5 (Abcam), pSTAT5 (Abcam), and tubulin (cell signaling).

### Cell viability

Cells were washed three times and resuspended in RPMI 1640 medium supplemented with 10% FBS, 100 units/ml of penicillin, and 100 μg/ml of streptomycin. To measure cell viability, 10,000 cells were seeded per well in 96-well plates. For each cell line, cells were treated with either FLT3 ligand (100 ng/ml), 10 ng/ml IL3, or no ligand. Following incubation for 48 h, cell viability was evaluated using AlamarBlue (Molecular Probe) according to the manufacturer’s protocol.

### Apoptosis

Cells were washed three times and resuspended in RPMI 1640 medium supplemented with 10% FBS, 100 units/ml of penicillin, and 100 μg/ml of streptomycin. One hundred thousand cells were seeded per well in a 12-well plate. Each cell line was treated with either FLT3 ligand (100 ng/ml), 10 ng/ml IL3, or no cytokine. After incubation for 48 h, apoptotic cells were measured by flow cytometry using annexin V and 7-aminoactinomycin D (7-AAD) apoptosis kit (BD Biosciences). Cells positive for annexin V and both annexin V/7-AAD were counted as apoptotic cells.

### Colony formation

Cells washed three times and resuspended in 20% IMDM medium and 80% methylcellulose medium (Stemcell Technologies). Five hundred cells were seeded in a 24-well plate and cultured for 7 days before counting colonies.

### FLT3 degradation assay

Cells were washed three times with PBS to remove cytokines, followed by incubation with 100 μg/ml cycloheximide for 30 min. Cells were then stimulated with FLT3 ligand (100 ng/ml) for 30 min in presence of cycloheximide, followed by lysis of the cells. Cell lysates were subjected to the SDS-PAGE and Western blotting analysis.

### Statistical analysis

All statistical analyses were performed using the unpaired, two-tailed Student’s *t* test with *p* < 0.05.

## Results

### Higher proliferation and survival of cells expressing TKD-ITD compared to cells expressing D835Y

**Fig. 1:**
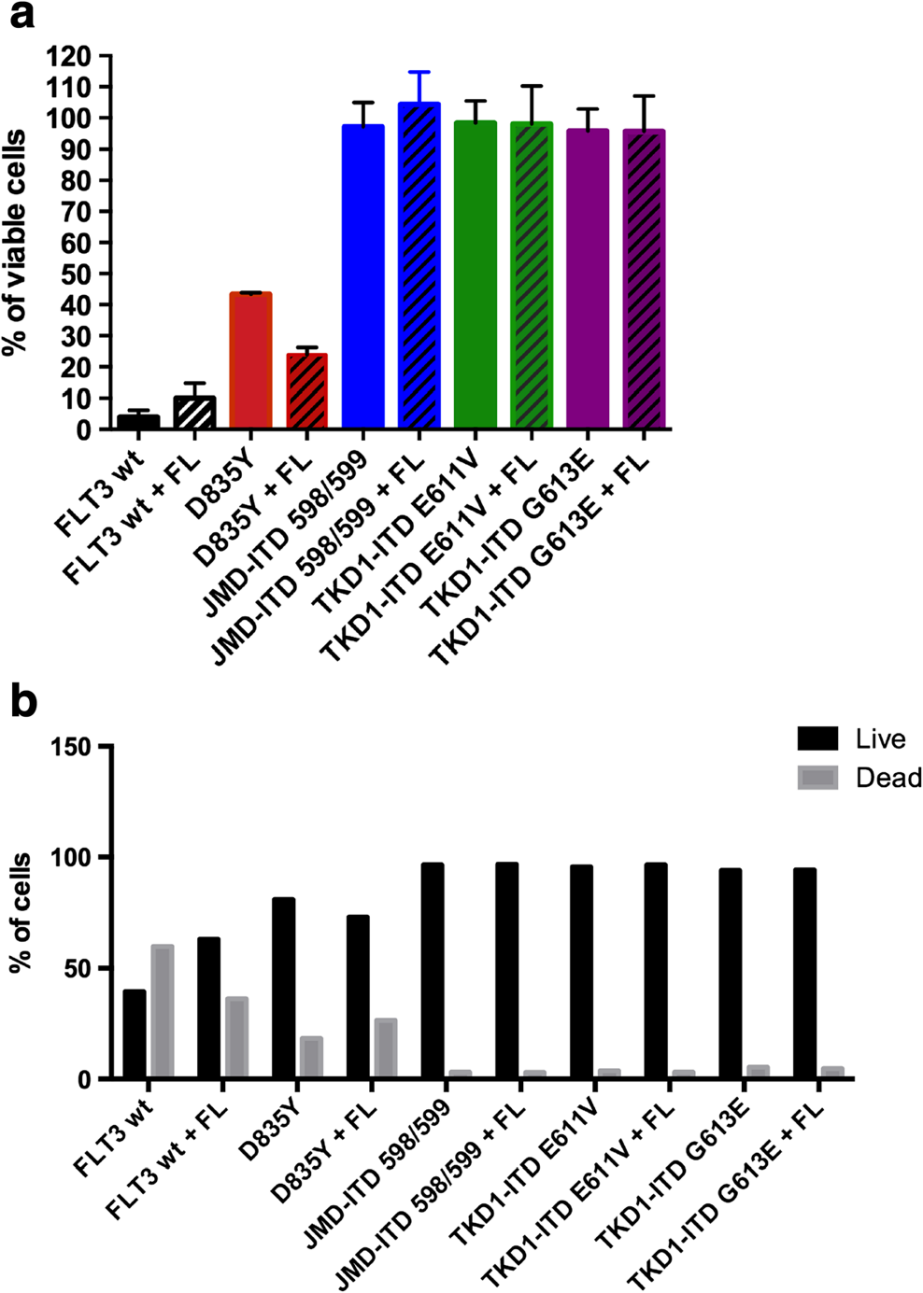
Higher proliferation and cell survival in TKD-ITD mutants compared to D835Y. **a** Cells were cultured in the absence or presence of FL (100 ng/ml) and normalized against cell viability in the presence of IL-3. Forty-eight hours post-seeding, cell viability was assessed using the AlamarBlue cell viability assay. Each bar represents the mean ± SD of a representative triplicate experiment. **b** Apoptosis was measured using annexin V and 7-AAD kit after 48 h of cytokine depletion

The ability of the cell to evade growth suppression and to divide uncontrollably, as well as its resistance to apoptosis, are some of the hallmarks of cancer [11]. Therefore, we first addressed the question whether there are any differences in viability and apoptosis between the recently identified ITDs—E611V (32) and G613E (33) [8]—and the most common point mutation D835Y, also located within the TKD. Ba/F3 cells stably expressing WT FLT3, D835Y, JMD-ITD 598/599 (22), TKD-ITD E611V (32), and G613E (33) [8] were used to investigate cell viability and apoptosis. An equal number of Ba/F3 cells were plated in three different groups: in the presence or in the absence of IL-3, as well as with the addition of FLT3 ligand (FL). The cells were cultured for 48 h and analyzed by PrestoBlue cell viability assay. WT FLT3 cells, grown in the presence or absence of IL-3, were used as positive and negative controls, respectively. As expected, there was no difference in cell viability in the presence of IL-3 in the cell lines expressing either form of FLT3. We observed that while TKD-ITD, regardless of location, can fully support viability in the absence of cytokines, D835Y-expressing cells displayed reduced viability (Fig. 1a). We also observed a significant increase in apoptosis in cytokine-starved cells expressing the D835Y mutant compared to TKD-ITD-expressing cells (Fig. 1b). Taken together, these results indicate that ITD mutations, regardless of their location in the gene, can support ligand-independent cell viability, whereas the D835Y mutation can only partially support cell viability.

### Differences in downstream signaling between ITDs and the D835Y mutation

**Fig. 2:**
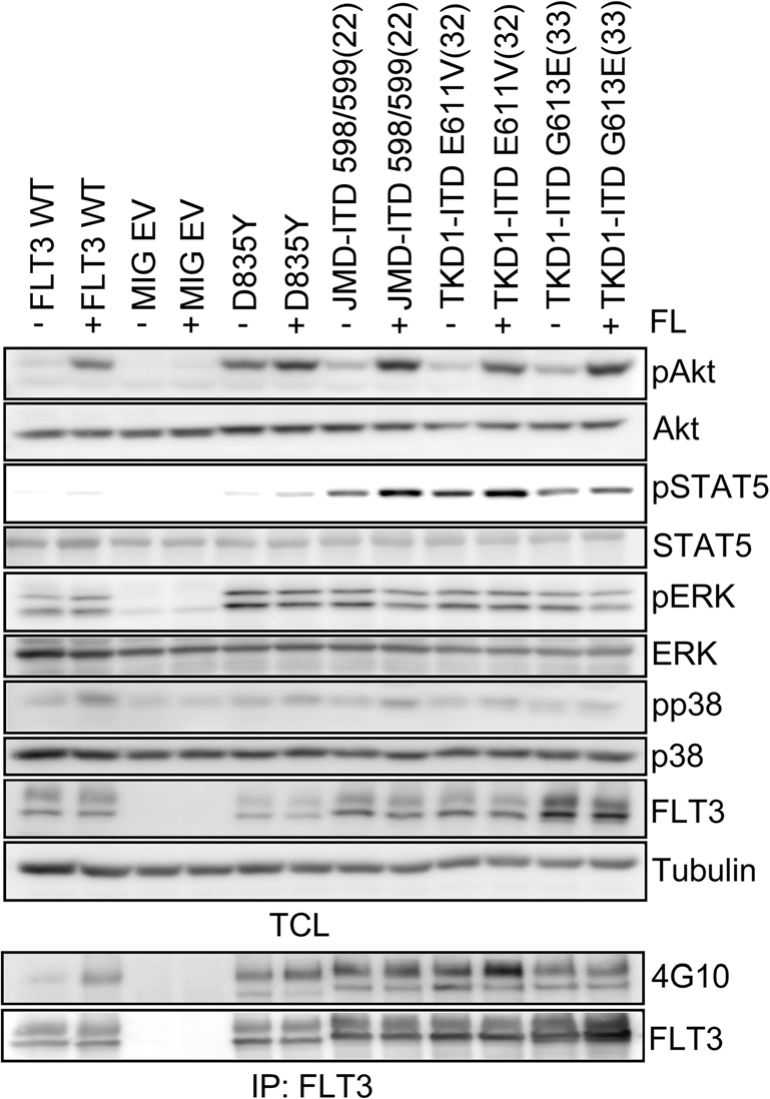
Activation of downstream signal transduction pathways by the different oncogenic mutants of FLT3 Ba/F3 cells were starved in serum- and IL-3-free media for 4 h before stimulating with 100 ng/ml of FL for 5 min. Cells were then lysed and lysates were used for SDS-PAGE and Western blotting analysis. The membrane was probed for different downstream signaling proteins and their phosphorylated forms

To investigate the underlying mechanisms that could explain the differences in phenotype between cells expressing ITDs and D835Y located within the tyrosine kinase domain, we compared several signaling pathways downstream of FLT3. For this purpose, Ba/F3 cells expressing either ITD-598/599 (at the JMD; 22), ITD-E611V (32) and ITD-G613E (33) (at the TKD), or D835Y were starved of IL-3 and serum for 4 h, followed by 5-min stimulation with FLT3 ligand (FL). As controls, Ba/F3 cells stably expressing WT FLT3 or empty vector (MIG EV) was used. Cell lysates were prepared and subjected to immunoblotting using specific antibodies against signal transduction intermediates. Previous studies have shown that ITDs at the JMD, but not the point mutation D835Y in the TKD, can induce tyrosine phosphorylation of STAT5 [12, 13]. We observed a similar pattern between the point mutant and ITDs located within the TKD (Fig. 2). However, cells expressing D835Y showed very weak tyrosine phosphorylation of STAT5, whereas TKD-ITDs showed strongest STAT5 phosphorylation (Fig. 2). In a striking manner, constitutive activation of AKT in Ba/F3 cells expressing D835Y was stronger compared to the ITDs (Fig. 2). Meanwhile, the phosphorylation levels of ERK and p38 remained at the same level in cell lines expressing either D835Y or ITDs. The tyrosine phosphorylation of FLT3 did not show any difference between different FLT3 mutants, except for TKD-ITD E611V (32), where we could observe increased ligand-induced phosphorylation of the receptor (Fig. 2).

### The TKD-ITDs and D835Y display different transforming potential

**Fig. 3:**
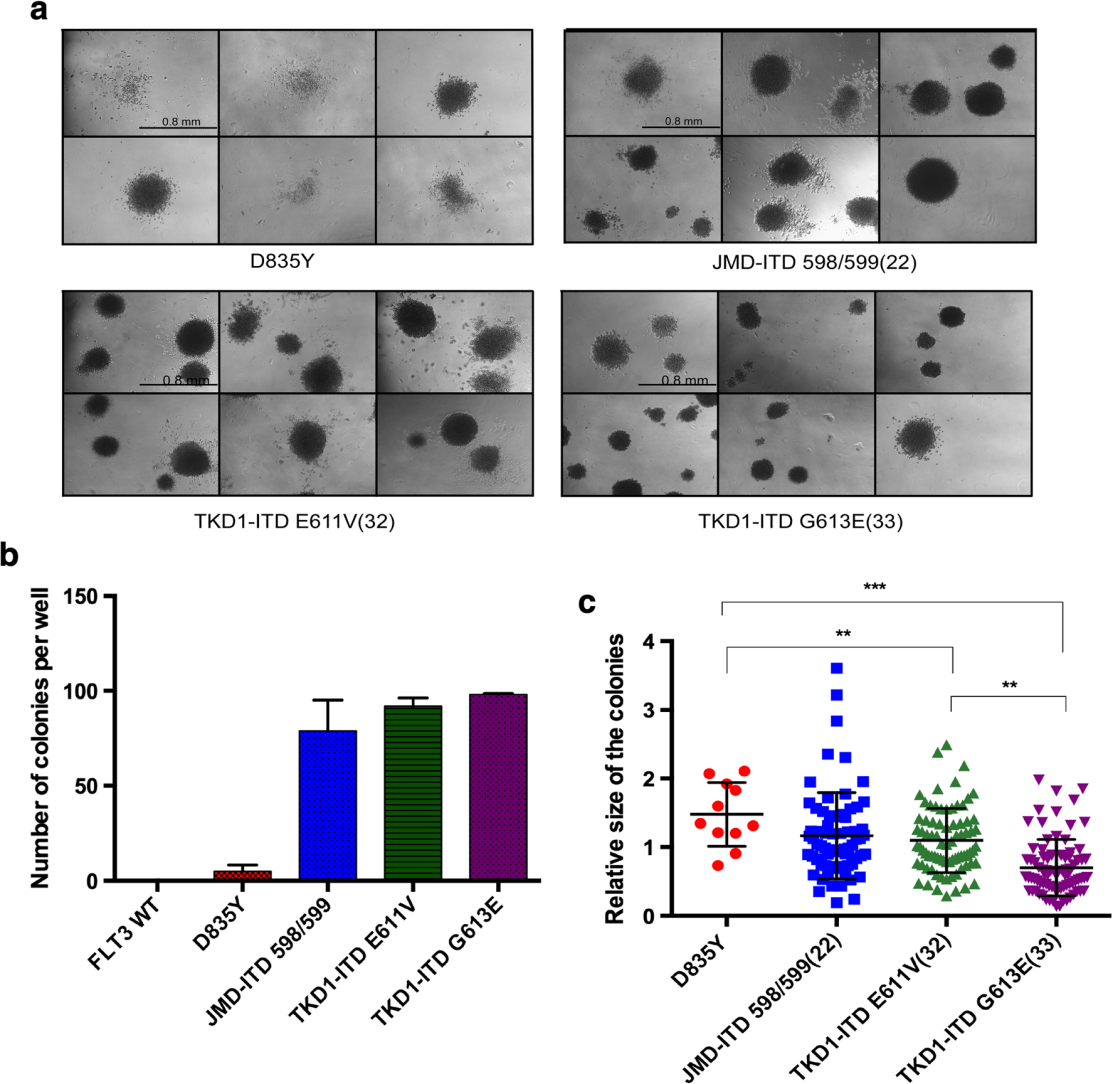
Both JM-ITD and TKD-ITD induce clonogenic growth in semisolid media in the absence of IL-3, while D835Y fails to induce colonies. The Ba/F3 cells stably expressing the indicated FLT3 mutants were plated in triplicate at a concentration of 500 cells per well. The plates were cultured for 7 days. **a** Photographs of the representative areas of the wells demonstrating the number of colonies. WT FLT3-expressing Ba/F3 cells failed to induce colonies (data not shown) **b** Quantification of the number of colonies per plate. Each bar represents the mean ± SD of a representative triplicate experiment. **c** The total area of each colony was analyzed with help of ImageJ. The data presented indicate the relative size of the colonies (*p value = *p* < 0.05)

To further analyze the transforming potential of ITDs and D835Y, the colony-forming capacity in cytokine-free methylcellulose medium was evaluated. The cells were washed to remove IL-3 and cultured in methylcellulose medium for 7 days, and the number of colonies was counted. As expected, WT FLT3 cells deprived of IL-3 failed to form any colonies. Ba/F3 cells expressing ITD mutants showed similar number of colonies regardless of ITD location, in agreement with a previous report [8]. In contrast to TKD-ITD, the expression of D835Y led to a significantly decreased number of colonies (Fig. 3a, b). Along with a count of the number of colonies, the area of each colony was also measured. Despite a lower number of colonies formed by D835Y cells, the areas of the colonies were larger, but not as evenly shaped as those formed by ITDs (Fig. 3c). Inspecting the cells expressing TKD-ITD-E611V (32) and TKD-ITD-G613E (33), we observed no change in the number of colonies, although the size of the colonies differed (Fig. 3c). We can conclude that ITDs at the TKD are capable for growth factor-independent proliferation and clonal growth of single cells, whereas the D835Y point mutant is more similar in its phenotype to ligand-activated WT FLT3. Overall, these results show that the transforming capacity of the TKD-ITD mutations is stronger than that of the D835Y mutant.

### Regardless of the nature of the activating FLT3 mutations, the stability of FLT3 remains unaltered

**Fig. 4:**
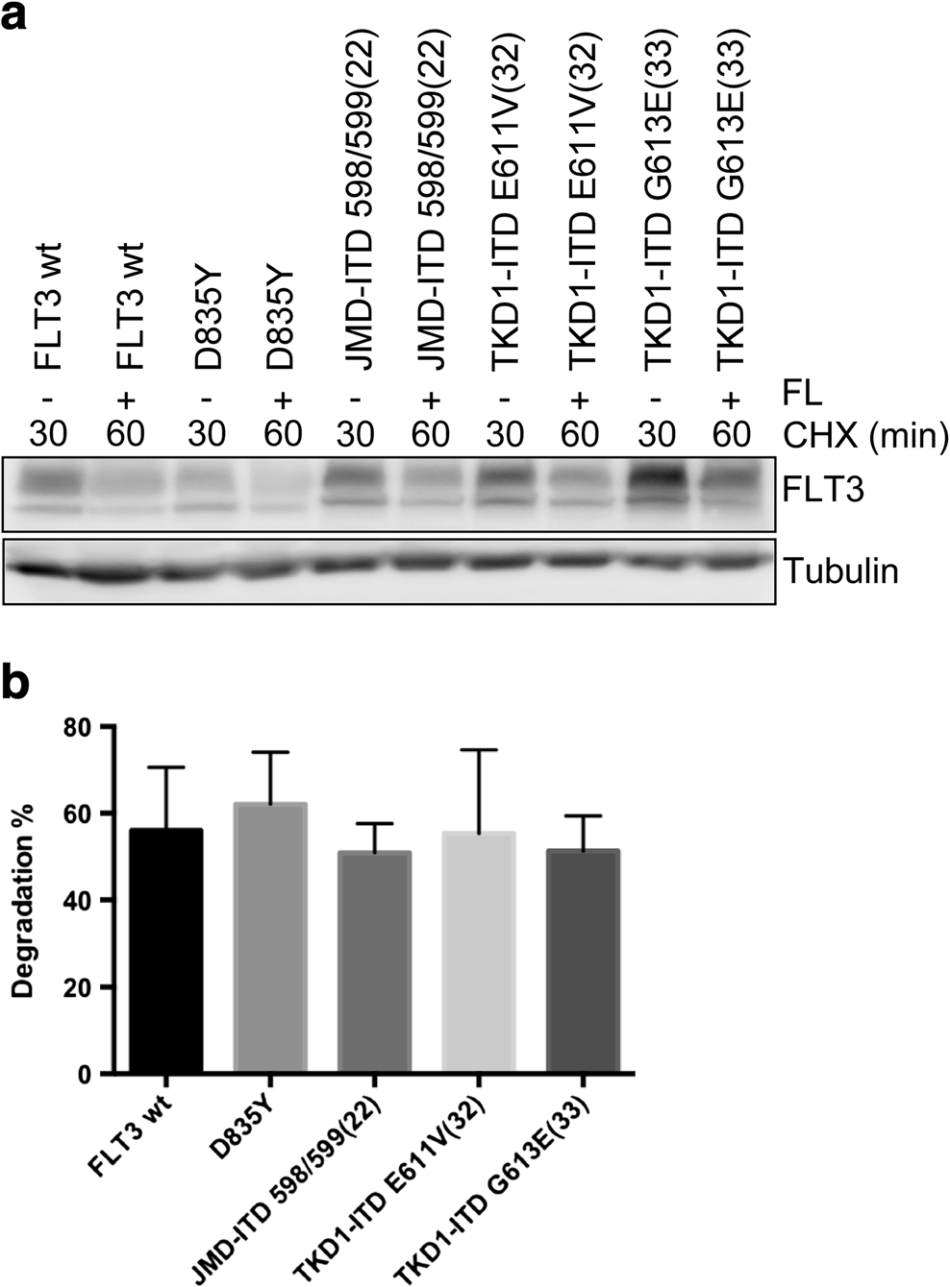
Stability of the various FLT3 mutants. **a** Cells were treated with cycloheximide for 30 min and lysed or stimulated with FL (100 ng/ml) for additional 30 min. Western blotting analysis was used to measure the degree of degradation. **b** The graph presents a summary of quantified degradation of the FLT3 receptor. Each bar represents the mean ± SD of a representative triplicate experiment

Finally, we analyzed whether the point mutation D835Y within TKD or ITDs within JMD or TKD have any effect on FLT3 stability. Ba/F3 cells were treated with cycloheximide for 30 min in order to stop protein synthesis, followed by immediate lysis of the cells or by stimulation with FL for additional 30 min in the presence of cycloheximide. We did not observe any differences in FLT3 degradation (Fig. 4a). In Fig. 4b, the quantification and normalization of three independent Western blots indicated no significant change. Therefore, the stability of the FLT3 receptor is not influenced by the nature of the activating mutations.

## Discussion

Since the discovery of FLT3 mutations, they have been intensively studied both regarding their molecular biology and their clinical relevance. Over the past years, new generations of tyrosine kinase inhibitors were developed; nevertheless, the relapse of patients with AML remains an undeniable problem. The number of relapsed patients with D835 point mutations is relatively low, suggesting that FLT3 point mutations are not involved in the development of the relapse [14]. In contrast, patients with the ITD mutations are associated with higher rates of relapse and poor overall survival [15, 16]. The purpose of this study was to directly compare recently characterized ITDs within TKD and the well-known point mutation D835Y within TKD, to understand whether the mutation’s nature or location plays the driving role in leukemogenesis. All three types of FLT3 mutations result in the constitutive ligand-independent activation; nevertheless, the downstream players and transformation capabilities are different.

In spite of mutations being located within the tyrosine kinase domain, only ITDs are capable of activating STAT5. It is likely that the TKD-ITDs localizes in the same manner, as was previously reported for JMD-ITDs, to the endoplasmic reticulum (ER) where it aberrantly activates STAT5 [17]. It has been shown that STAT5 activation by ITDs located within JMD domain is mediated through the SRC family kinases [18]. It was also shown that phosphorylation of the SRC binding sites in FLT3, Y589 and Y591, were phosphorylated to a higher extent in FLT3-ITD compared to FLT3/D835Y, which could explain the stronger STAT5 phosphorylation in FLT3-ITD compared to FLT3/D835Y. It is not completely known which SRC family members are involved in phosphorylation. We have previously demonstrated that both FYN and LCK can positively contribute to FLT3-ITD-mediated STAT5 phosphorylation [19, 20]. Regardless of the mechanism, the aberrant activation of STAT5 is an essential step in myeloid transformation [21, 22]. Therefore, cells expressing ITDs independent of their location in the gene show a stronger phenotype in terms of activation of STAT5 and thus proliferation rate and anti-apoptotic activity in comparison to D835Y.

Some signaling pathways, such as the phosphorylation of ERK and p38, were very similar between the various FLT3 mutants. In contrast, cells expressing the D835Y mutant of FLT3 displayed constitutive AKT activation, whereas the ITD mutants displayed stronger ligand-induced AKT phosphorylation. The reason for this discrepancy is not completely clear. We know that the D835Y mutation not only leads to constitutive activity of FLT3, but that the cells expressing D835Y are independent of SRC kinase activity for transformation [18]. This might be explained by altered kinase specificity of the D835Y mutant compared to wild-type FLT3. In the closely related KIT, mutation of the analogous site D816 to valine leads not only to constitutive activity of the receptor, but also to an altered kinase specificity resembling the kinase activity of SRC family kinases [23]. The reason for ligand-induced AKT phosphorylation is not clear. It seems counter intuitive that a mutant that is trapped in the ER could have the ability to induce AKT activity in a ligand-dependent manner. However, even though a majority of FLT3-ITD remain trapped in the ER, there is also FLT3-ITD expression on the cell surface and some signaling pathways are activated from the cell surface, such as activation of KRAS [24]. Activation of PI3-kinase/AKT by FLT3 is known to occur through the scaffolding protein GAB2, which is phosphorylated by SRC family kinases and the subsequent recruitment of PI3-kinase to GAB2 [25, 26]. Thus, it is likely to be the mechanism behind the stronger ligand-induced AKT in the ITD mutants.

Degradation of receptor tyrosine kinases has been shown to be regulated by CBL-mediated ubiquitination, leading to internalization and degradation of the receptors. It is known that CBL-mediated ubiquitination in many cases is regulated by SRC-mediated tyrosine phosphorylation [27]. Thus, we hypothesized that there might be a difference in the stability of FLT3 depending on the nature of the oncogenic mutation. However, we found no difference in stability between the various oncogenic mutants of FLT3.

The presented results raise interesting questions about the role of the location and the nature of the mutation in the molecular pathogenesis of AML. In this study, we chose to only compare TKD-ITDs to FLT3/D835Y. However, it should be noted that, despite D835Y being the most frequent point mutation in AML patients, several other activating mutations have been reported in this region including other substitutions of D835 and small deletions or insertion mutations. Furthermore, the ITD mutations are located to the first part of the tyrosine kinase domain, whereas the FLT3/D835Y mutation is localized to the second part of the TKD, after the so-called kinase insert. One of the characterized FLT3-ITD mutations is located within the second part of TKD region (A627E), sharing a similar location to the point mutation D835Y [5].

In this study, we have used a specific FLT3-ITD sequence (N51; [28]); however, the sequence and the length of ITD mutations is extremely heterogeneous and varies from patient to patient [29]. In previous studies, it has been reported that in patients with FLT3-ITD mutations, the length of the ITD may change after therapy [30]. Additionally, a longer ITD size is associated with shorter overall and relapse-free survival and it has been suggested that FLT3-ITD size has a prognostic significance in AML [31]. Therefore, comparison of different ITD mutants will provide information on why the length of the ITD is important for patient survival. Taken together, our current study suggests that in the BaF3 cell model, the location of ITD mutations is of minor importance for transformation and that the D835Y mutation has a weaker transforming capacity than ITD in the TKD. In a previous study, it was found that FLT3-ITD location influences disease biology in vivo and leads to changes in global gene expression. ITD location altered proliferative capacity and sensitivity to FLT3-TKI treatment in vivo [8]. Therefore, it will be of major interest to study sensitivity of the FLT3-mutated clones and response rates to FLT3 inhibitor therapy in patients harboring JM-ITD versus TKD-ITD mutations.
